# A radiosensitivity MiRNA signature validated by the TCGA database for head and neck squamous cell carcinomas

**DOI:** 10.18632/oncotarget.5299

**Published:** 2015-10-06

**Authors:** Ning Liu, Rebecca J. Boohaker, Chunling Jiang, James R. Boohaker, Bo Xu

**Affiliations:** ^1^ Department of Oncology, Southern Research Institute, Birmingham, AL 35205, USA; ^2^ Department of Gastric Cancer Surgery, Tianjin Medical University Cancer Institute and Hospital, National Clinical Research Center for Cancer, Key Laboratory of Cancer Prevention and Therapy, Tianjin 300060, China; ^3^ Nanchang University Graduate School, Nanchang 330047, China; ^4^ Department of Radiation Oncology, Jiangxi Cancer Hospital, Nanchang 330029, China; ^5^ Department of Economics, American University, Washington, DC 20016, USA; ^6^ Cancer Cell Biology Program, Comprehensive Cancer Center, University of Alabama at Birmingham, Birmingham, AL 35205, USA

**Keywords:** miRNA, radiosensitivity, ATM

## Abstract

MicroRNA, a class of small non-coding RNAs, play critical roles in the cellular response to DNA damage induced by ionizing irradiation (IR). Growing evidence shows alteration of miRNAs, in response to radiation, controls cellular radiosensitivity in DNA damage response pathways. However, it is less clear about the clinical relevance of miRNA regulation in radiosensitivity. Using an *in vitro* system, we conducted microarray to identify a miRNA signature to assess radiosensitivity. The data were validated by analyzing available Head and Neck Squamous Cell Carcinoma (HNSCC) samples in the cancer genome atlas (TCGA) database. A total of 27 miRNAs showed differential alteration in response to IR in an Ataxia-Telangiectasia Mutated (ATM) kinase-dependent manner. We validated the list and identified a five miRNA signature that can predict radiation responsiveness in HNSCC. Furthermore, we found that the expression level of ATM in these patients was correlated with the radiation responsiveness. Together, we demonstrate the feasibility of using a miRNA signature to predict the clinical responsiveness of HNSCC radiotherapy.

## INTRODUCTION

Tumor responses to radiotherapy are influenced by many factors, including those of genetic, pathological, and tumor micro-environmental. For example, intra-tumoral hypoxia is one of the factors that contribute to radioresistance [1]. In recent years, efforts have been focused to identify molecular elements in regulating tumor radiosensitivity, and gene products that are involved in the DNA damage response (DDR) have been found to be associated with radiosensitivity [2]. However, it is still not clear how these DDR elements contribute to radioresistance in the tumor setting, and there is clearly a lack of clinical significance of these factors.

As a class of critical elements in the DDR, microRNAs (miRNAs) have been implicated in regulation of radiosensitivity [3, 4]. miRNAs are small non-coding RNAs that are involved in biological processes, including development, proliferation, cell-cycling and DNA damage repair [5]. These 20–25 nucleotide RNAs are processed from larger nucleotide fold-back structures (pri-RNA) into hairpin pre-miRNAs before finally being converted into mature miRNAs. The number of unique, mature human miRNAs reported is 2,588 (according to the June 2014 release of miRBase 21). The regulation of miRNA expression can be governed by a number of internal and external factors, and these factors uniquely regulate the maturation of specific miRNAs [6].

The differential expression of miRNAs in human tumors has led to the idea that these miRNAs can contribute to oncogenesis by acting as tumor suppressors or oncogenes. These changes in expression can promote genetic instability by negatively regulating DDR proteins, resulting in a decrease in the DNA repair capacity of cancerous cells [7, 8]. Studies have implicated a hypoxic tumor micro-environment and the increased expression of HIF-1α in the up-regulation of two specific miRNAs, miR-210 and miR-373. The expression of these two miRNAs was found to specifically alter the nucleotide excision repair and homology-dependent repair pathways [9]. Similarly, exposure to ionizing radiation (IR) results in an altered miRNA expression profile [10]. Alterations in expression become critical in terms of radiosensitivity, because activation of the DDR, in an Ataxia-Telangiectasia mutated (ATM) kinase-dependent manner, provide regulatory targets for miRNAs expressed in the presence of IR at each stage of the checkpoint [10]. Internal regulation and maturation of specific miRNAs induced by DNA damage can be linked to activation of ATM, and the subsequent phosphorylation of the KH-type splicing regulatory protein (KSRP) [11].

The determination and characterization of miRNA expression profiles, particularly in cancer subtypes, can play an important role in understanding disease progression and treatment responses. Examination of miRNA expression profiles in both laboratory and clinical settings might establish miRNA “fingerprints” that correlate to, among other parameters, cancer type, aggression, and response to treatment. In Head and Neck Squamous cell carcinomas (HNSCC) for example, a comparative analysis of normal and cancerous tissues revealed that a handful of miRNAs were differentially regulated in the cancerous tissues with one in particular, Mir-21 as a putative oncogenic miRNA [12]. Most recent studies underscoring the robustness of The Cancer Genome Atlas (TCGA) database utilized the database to annotate and analyze somatic variants in HNSCC patients [13]. Here we focus specifically on the effects of ionizing radiation on miRNA expression levels, and the correlation of the expression to radiosensitivity to identify a miRNA signature for the predication of radiosensitivity.

## RESULTS

### Ionizing radiation-induced miRNA changes in cell lines with proficient or deficient of ATM

In order to assess the relationship of miRNA changes with radiosensitivity, we selected a pair of human lymphoblastoid cell lines derived from a patient (proband) of Ataxia-Telangiectasia and a direct relative (normal). These two cell lines have been extensively used to study the functional role of the ATM kinase in the DDR previously [14–16]. We conducted a full microRNA array in cells treated with or without IR. To analyze the data, we first established the threshold fluorescence intensity for each of the four experimental conditions (GM0536 mock or IR and GM1526 mock or IR): ${\hat{\theta }}_{{}_{\text{GM}0536\text{R}}}=(203.33437;\;204.001085)$, ${\hat{\theta }}_{{}_{\text{GM}0563\text{C}}}=(187.334165;\;187.665806)$, ${\hat{\theta }}_{\text{GM}1526\text{R}}=(174.00038;\;174.33339)$, and ${\hat{\theta }}_{\text{GM}1526\text{C}}=(174.675988;\;175.000051)$. We then focused on identifying miRNA fold changes in response to IR, and the analysis returned 28 miRNAs that were significantly altered after IR in either the GM0536 or GM1526 cell line (Table 1). Of the identified miRNAs, 16 were differentially altered in the ATM^−/−^ GM1526 cell line, and of those, 12 were significantly up-regulated (≥ +2 standard deviations), with the other 4 significantly down-regulated (≤ −2 standard deviations) (Significance was determined to be a fold-change after radiation that was ± 2 standard deviations in the fold change of expression). The list of miRNAs was then defined as hits.

**Table 1:** miRNA targets identified from microArray screening

miRNA	Fold change (after IR) ATM+/+	± SD	Fold Change (After IR) ATM−/−	± SD
hsa-let-7e	0.89194	0.0388	1.42886	0.0335
hsa-let-7f	1.03497	0.1200	1.62202	0.0341
hsa-let-7g	1.20312	0.0359	1.07888	0.0470
hsa-mir-016	0.88144	0.1411	2.24896	0.1462
hsa-mir-019a	0.89458	0.0141	0.91168	0.0298
hsa-mir-019b	0.88981	0.0490	0.9242	0.0610
hsa-mir-020a	1.19119	0.1565	0.82158	0.1914
hsa-mir-027a	0.97308	0.0415	1.40207	0.0356
hsa-mir-029b	0.78003	0.0145	1.56938	0.1775
hsa-mir-029c	1.07076	0.1039	1.20673	0.1883
hsa-mir-030c	0.55318	0.0699	1.60287	0.1257
hsa-mir-030e 3p	1.05512	0.0711	0	0
hsa-mir-101	0.92582	0.0323	0.73823	0.0517
hsa-mir-106a	0.80357	0.0579	1.92872	0.0678
hsa-miR-1248	2.2929	0.1513	0	0
hsa-miR-1254	0.75457	0.0154	0	0
hsa-miR-1308	0.85653	0.0533	1.06383	0.0970
hsa-mir-142 3p	0.79681	0.0180	0.83335	0.1186
hsa-mir-150	1.14678	0.0265	0	0
hsa-miR-1826	1.22729	0.0339	3.11047	0.0832
hsa-miR-18b	0.80097	0.0894	0.86415	0.0261
hsa-miR-320c	1.18737	0.0490	0	0.0368
hsa-mir-565-Pre	1.37948	0.0744	1.02796	0.0482
hsa-mir-566-Pre	0.59372	0.0217	1.29855	0.1471
hsa-mir-595	0	0	0	0
hsa-miR-768-3p	1.16511	0.0261	0	0
hsa-miR-768-5p	1.28797	0.0771	0	0
hsa-miR-886-5p	1.08908	0.0675	0.76209	0.0305

### Validation of the miRNA hits using the TCGA data base

**Figure 1:**
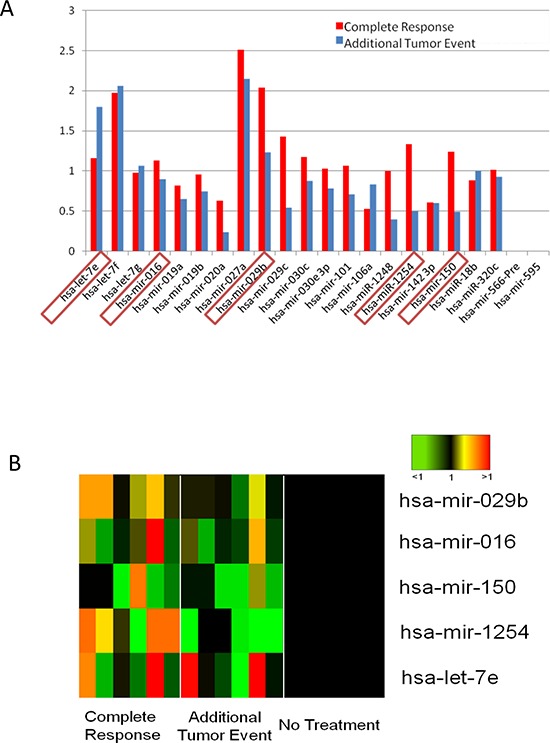
Differences in miRNA expression patterns between patients with complete response and those with additional tumor events **A.** Relative miRNA expression of the 22 miRNAs identified in the *in vitro* microarray was plotted. Fold change was cross referenced with the *in vitro* fold change. The boxed miRNAs positively correlate with *in vitro* data. Significance was determined by Student's *t*-test; *p ≤ 0.05, **p ≤ 0.005, ***p ≤ 0.0005 **B.** A heat map of the levels of the 5 correlating miRNAs in patients with complete response or additional tumor event relative to patients receiving no treatment indicates an increase in the event of a complete response.

To assess the clinical relevance of the 28 miRNAs hits, we accessed the TCGA database and focused on available cases of Head and Neck Squamous Cell Carcinomas. We compared the levels of each miRNA expression in patient samples. Of the 523 available patient cases in the database, 435 patients were monitored by clinical follow-up. These patients were monitored for either complete remission or additional tumor event, and were categorized as having radiation or chemotherapy. The clinical data for a majority of the patients was often incomplete, so we focused our analysis on those patients with the most complete set of criteria in terms of tumor status and course of treatment. Specifically, we chose a subset of cancer patients in which the primary course of treatment was radiotherapy. An analysis of three groups of patient miRNAs was performed, with each group comprised of 10 patients representative of their respective groups. The groups analyzed were as follows: radiated with a complete response (defined as radiosensitive), radiated with tumor progression (defined as radioresistant), and not irradiated. Highlighting the expression of those 28 miRNAs identified in the microArray screen, a ratio of the average expression levels in the patients receiving radiotherapy vs. those not receiving radiotherapy was calculated (Figure 1A). The ratios were then compared to the fold change values from Table 1. From the correlation of the patient data with the *in vitro* data, a subset of 5 miRNAs emerges as a signature for predicting radiation response in the clinical setting (Figure 1B). In this pattern, upregulation of miR-016, miR-29b, miR-150 and miR-1254, and down regulation of let-7e after radiation correlated with a complete response. On the contrary, an inverse expression pattern was seen to be associated with additional tumor events in the samples.

### ATM expression level in HNSCC is associated with the radiation response

**Figure 2:**
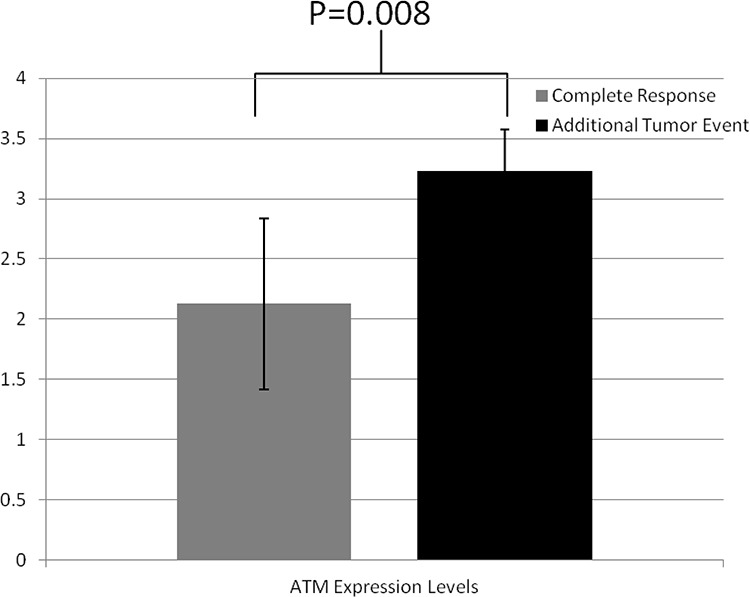
ATM expression levels are higher in patients with additional tumor events following radiation treatment RPPA data from the patients cataloged in the TCGA database shows that there is an increase in ATM protein levels in patients with additional tumor events.

As a central DNA damage response protein, ATM-deficiency leads to radiosensitivity [17]. ATM-mediated radiosensitivity is regulated by its phosphorylation of downstream targets such as SMC1 and Snail [18, 19]. To determine whether the various radiation responses in the HNSCC patients were linked to different ATM expression, we looked at the ATM expression level in the same patient population in the TCGA database. Protein expression for the TCGA HNSC samples was determined using MDA Reverse Phase Protein Array. The average levels of ATM expression from the radio-sensitive samples and radio-resistant samples were calculated. Interestingly, we found that ATM expression levels were significantly lower in the radio-sensitive patients when compared to the resistant patient levels (Figure 2).

### Verification of miRNA signature by qRT-PCR

**Figure 3:**
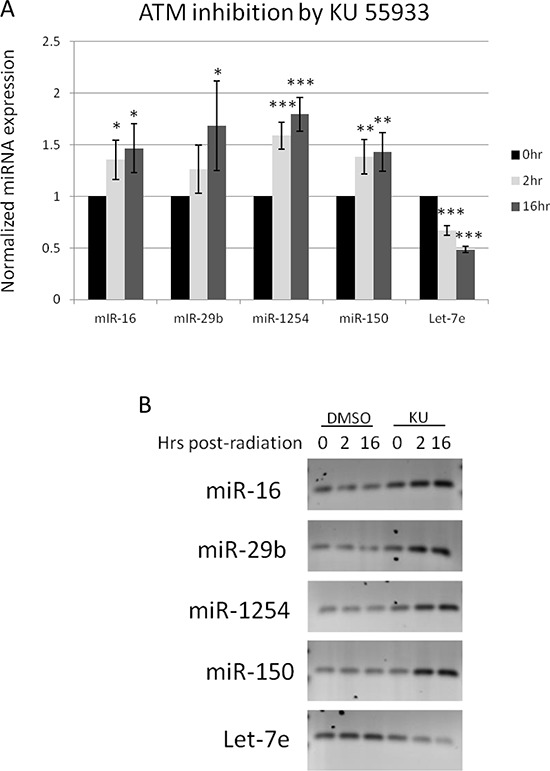
qRT-PCR of miRNA signature in HNSCC cells **A–B.** Cal27 cells were subjected to ionizing radiation in the presence or absence of the ATM kinase inhibitor KU-55933. The miRNA levels were quantified by qRT-PCR, the expression levels of each miRNA were normalized according to the Cq values. In the KU-55933 treated cells, there were significant changes in all 5 miRNAs probed as determined by Student's *t*-test for variance. *P* values are reported as *p ≤ 0.05, **p ≤ 0.005, ***p ≤ 0.0005.

In order to determine the validity of the identified miRNAs as a signature, we tested a head and neck cancer cell line Cal27, a tongue Squamous cell carcinoma line. The miRNA expression profile of the Cal27 before and after radiation, with and without ATM inhibition is consistent with both the initial miRNA screen and, more importantly, the TCGA clinical data (Figure 3A). As with the TCGA data and the initial miRNA screen, in the absence of ATM function, miR-16, −29b, −1254, and −150 were up-regulated, while Let-7e was down-regulated. To confirm the qRT-PCR, the amplified products were run on an agarose gel (Figure 3B). The results indicate that this identified signature represents radiosensitivity in HNSCC. A comparison of the basal miRNA levels with and without ATM inhibition indicates that ATM plays a minimal role in the basal regulation of these miRNAs (Supplemental Figure 1).

### A pathway analysis of the miRNA signature that predict radiation responsiveness

**Figure 4:**
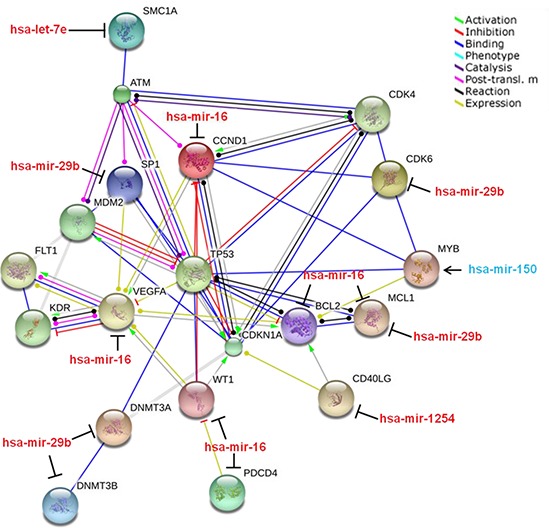
Pathway analysis of proteins regulated by the 5 “fingerprint” miRNAs String pathway analysis of the proteins regulated by the identified miRNA fingerprint links elevated expression of the 5 miRNAs with cell survival, cell cycle, and DNA damage pathways.

A pathway analysis of the targets regulated by these 5 signature miRNAs was determined using the String database (STRING 9.1) (Figure 4). Of the multiple gene targets of these miRNAs, a handful is implicated in the DDR. Interestingly, we found that SMC1A, a direct target of ATM in radiosensitivity [18], is regulated by let-7e by the pathway analysis. Conversely, BCL-2 and MCL1 are regulated by miR-16 and miR-29b respectively [20, 21], which we have observed to be up-regulated in radiosensitive patients with complete regression of the disease. The direct connection of the targets of the miRNAs to key proteins in the DDR, specifically ATM, MDM2 and TP53, indicated that the expression patterns of these miRNAs present a viable biological fingerprint for radiosensitivity phenotypes.

## DISCUSSION

Identification of a miRNA signature to predict tumor response to radiotherapy can provide a more comprehensive picture as to how miRNAs are involved in the radiation response. With the correlation of this signature in the TCGA database, we demonstrate a clinically relevant biomarker tool that might provide useful information to help make a treatment decision. For example, if the radiation-induced miRNA signature change can be obtained after the first week of radiation treatment, and analyzing the data might determine whether a booster dose of radiation is needed by the end of radiotherapy course.

The ability to link *in vitro* observations to clinical relevance relies on the curation of a patient database. The TCGA network provides a large scale view of numerous parameters for cancers in nearly a dozen tissue types. The public availability of this data allows for the reconciliation of non-clinical data with information gathered from patients in order to more effectively translate results from the bench into practical clinical application. Recently, it was shown that the patient repository of the TCGA database can be utilized in a disease specific manner to highlight parity in somatic mutations that can be leveraged into advances in treatment of HNSCC patients [13]. The database parameters examined here focused on miRNA and protein expression levels, and disease progression of patients with HNSCC. The radiosensitivity miRNA signature identified was validated by the clinical data, indicating that miRNAs can be used as biomarker to predict clinical responsiveness to radiotherapy.

Previous mechanistic studies have provided clues on how these 5 miRNA signature proteins are involved in regulating radiosensitivity. Specifically, pathway analysis shows that identified signature miRNAs regulate mRNA levels of proteins involved in pro-survival pathways and DNA repair pathways. Using differential miRNA expression as a means for establishing a fingerprint or biomarker has the potential to be a reliable prognostic tool, particularly if the absence of a functional ATM results in an elevated basal expression of these miRNAs. The miRNAs that were identified in this study have been implicated in disease progression in a number of cancer types; however their expression has not be examined in the context of radiation treatment.

By considering the 5 miRNAs as a signature as well as the correlation of ATM expression with radiation resistance we can begin to validate two means for predictive biomarkers: 1) Prior to radiation, ATM expression can be assessed. Based on the TCGA data as well as the initial microarray screen, the direct correlation between ATM levels and radio-resistance can be used to determine the effectiveness of radiotherapy. 2) A post-radiation assessment of our target miRNA signature expression early in the radiation course can be predictive of the sensitivity of the tumor to the treatment. If the signature indicates a resistance, as determined by the relative expression levels defined above, then a booster radiation dose may be required for a complete tumor response.

## MATERIALS AND METHODS

### Cell lines and culture

The Epstein-Barr virus transformed human lymphoblastic cell lines GM0536 and GM1526 cells (obtained from the NIGMS Human Mutant Cell Repository, Camden, NJ) were maintained in RPMI 1640 medium supplemented with 15% fetal bovine serum and 50 μg/ml of penicillin/streptomycin. HNSCC CAL 27 cells (CRL-2095, ATCC Manassas, VA 20108 USA) were maintained in DMEM medium supplemented with 10% FBS containing Pen/Strep antibiotics in a humidified 37°C atmosphere. Cell line authentication was originally done by Short Tandem Repeat profiling at NIGMS and ATCC, respectively. After purchase, the cell lines were not authenticated by authors.

### Irradiation

Ionizing radiation was delivered by an X-Rad 320 X-ray irradiator (Precision X-Ray Inc. N Branford, CT) at a dose rate of 2Gy/min.

### MiRNA Microarray analysis

MiRNA microarray analyses were performed by GenoSensor (Tempe, Arizona). 2 hours after 0Gy or 6Gy irradiation, cells were harvested and the total RNA isolated followed by miRNA microarray. Genes are positioned by array block, row, and column. All genes are in triplicate. Their names and signal intensity of each probe were directly transferred from the raw data sheets. The average of signal intensity on three redundancies and standard deviation were calculated. Signal intensity is fluorescent signal of each chemical. Since the fluorescence dye is detected at either 635 nm or 532 nm channel, the signals are labeled with either F635 or F532. Bacterial sequences, such as gnd, fixB, etc., were served as negative controls and for assay QA/QC analysis, the sequences of which are not homologous with any targets in the reaction. Housekeeping genes are used as positive controls, such as U6 and 5S-rRNAs. Normalization is represented by the ratio of the average of gene signal intensity of a specific gene vs. average of signal intensity of all positive control genes. Fold change is comparison between samples, calculated by the ratio of normalization value.

### Quantification of miRNA

Total RNA was isolated (Trizol Reagent, GIBCO BRL) at 0, 2, and 16 hrs of Cal27 cells post radiation (5Gy IR) treated with and without KU 55933 (ATM inhibitor, Catalog number 3544, Tocris, Bristol, BS11 0QLUnited Kingdom), and cDNA was generated using All-in-One™ miRNA qRT-PCR kit (GeneCopoeia, Rockville, MA 20850 USA). The miRNA levels of 5 target miRNAs were measured by qPCR at miRNA expression levels were compared to those of cells treated with KU 55933 (4 μM Reverse transcription was performed on the Arktik thermal cycler (Thermo) according to the manufacturer's protocol. The cDNA was then mixed with respective miRNA primer probes (GeneCopoeia). The plates were run on the BioRad CFX-96 plate reader. Fluorescence was read in the green channel. Cq values were averaged and compared to positive and non-template controls. The quality of the amplified cDNA was determined by a melt curve at the end of the cycle. Amplification was verified by running 10 μl of the PCR products on a 3% agarose gel. Gels were imaged on an AlphaInnotech FluorChemHD2 gel dock. Changes in miRNA expression were analyzed for significance using the Student's *T*-test for variance. The experiment was repeated twice with samples in triplicate each time.

### Statistical analysis

In running a logistic regression model, we found that the independent variable was a perfect predictor for the dependent variable. Hence, it follows that a threshold analysis would be the best course of action. A threshold analysis was performed on the fluorescence values reported from the microRNA array of the two cell lines under the two experimental conditions. The threshold analysis, $\exists \theta \in R^{+}$, is such that if X>θ then the result returns a success, and if X≤θ then the result returns a failure. $f:{\bar{R}}^{+}\to \{S,F\}$ when S is the event of a success and F is the event of a failure so that $f(X)=\{\begin{array}{c} S:x>\theta \\ F:x\leq \theta \end{array}$.

A fold change test was conducted on the data set as a whole using an if/then statement to separate successes from failures. Failures were microRNAs with fluorescence values that reported a zero from the fold change test. Successes produced *a* value greater than zero. Next, the interval for where the true threshold, *θ*, actually exists was determined. This was done by taking the minimum fluorescence from the successes and the maximum fluorescence from the failures to form an interval of interest. To do this, a new function was established: $g\;:\;R^{2}\to R^{2}$ where g(X|f(X)=S, X|f(X)=F)=max{X|f(X)=F}, min{X|f(X)=S}. Here, X|f(X)=S is the data set where the fluorescence levels returned a positive value from the fold change test, and X|f(X)=F is the data set where the fluorescence levels returned a zero from the fold change test. From this function the threshold values for each experimental condition were determined.

Power analysis for the selection of patient data per condition was determined using the following equation:

$$n=\left(\frac{8.52}{{\left\{\frac{1}{2}\left(\ln\left[1+R\left(y,x\right)\right]\right)-\ln\left[1-R(y,x)\right])\right\}}^{2}}\right)+3$$

### TCGA database analysis

Archived patient samples of HNSCC patients in The Cancer Genome Atlas (TCGA) database (https://tcga-data.nci.nih.gov) were selected based on patient samples that had been subjected to both miRNA-Seq testing and reverse-phase protein array (RPPA) analysis. The data from 523 patients was available through the database. The miRNA levels of the specific miRNAs identified in the microarray from the *in vitro* study were examined and averaged in these patients. The ATM protein levels of the patients were averaged from the RPPA data available. Patients were able to be separated according to response to treatment based on the follow-up data also archived within the database.

### Pathway analysis

The miRNAs identified from patient and *in vitro* screens were then input into the miRecords database (http://mirecords.umn.edu/) to determine validated target mRNAs for these specific miRNAs. The protein hits from miRecords were then collectively input into the STRING functional protein association networks (http://string-db.org/) to determine functional protein interactions.

## SUPPLEMENTARY MATERIALS FIGURE


