# Supplementary material for: A radiosensitivity MiRNA signature validated by the TCGA database for head and neck squamous cell carcinomas
Source: Oncotarget. 2015 Oct 6;6(33):34649–57. doi: 10.18632/oncotarget.5299 (PMC4741479; doi:10.18632/oncotarget.5299)
Supplement: Supplementary file 1 [file oncotarget-06-34649-s001.pdf]

SUPPLEMENTARY FIGURE

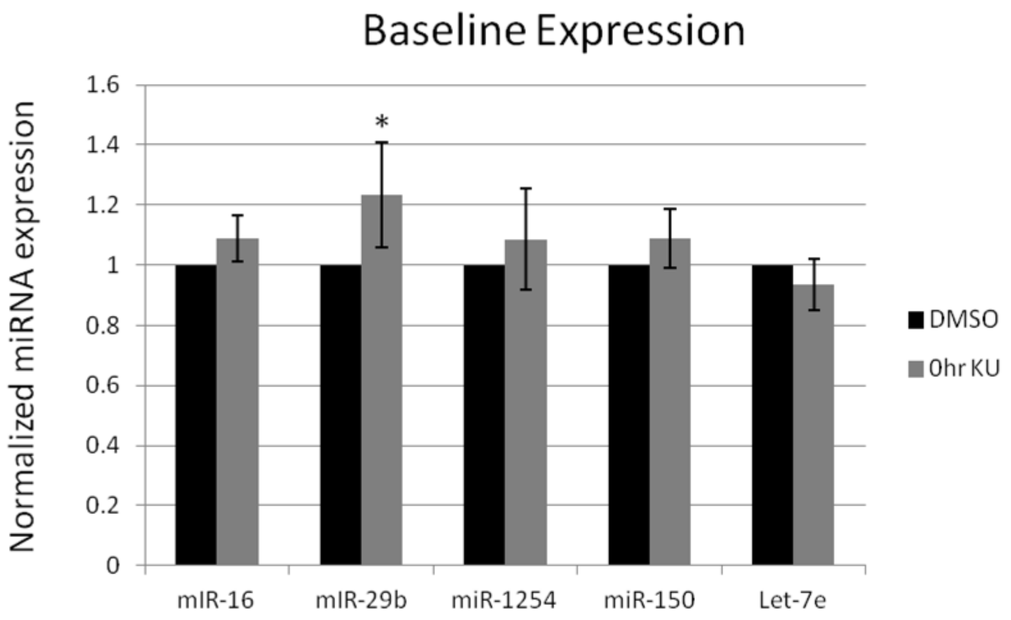

Supplementary Figure S1: Baseline expression of signature miRNAs. Cal27 cells were treated with an ATM kinase inhibitor (KU treated) or DMSO as control. The miRNA levels were quantified by qRT-PCR, the expression levels of each miRNA were normalized according to the Cq values. KU treated cells, showed slightly elevated levels of miRNA when compared to the DMSO as determined by Student's *t*-test for variance. The only significantly elevated basal level was miR-29b with a *P* value reported as $*p \leq 0.05$.
